# Introductory

**Published:** 1861-08

**Authors:** 


### EDITORIAL DEPARTMENT.

### INTRODUCTORY.

In introducing to the medical profession the first number of the *Buffalo Medical and Surgical Journal and Reporter*, it may be expected that some reasons for its appearance will be given, and also some account of the objects to which it will be devoted, the aims and purposes it is designed to subserve.

With regard to the reasons for its appearance at the present time, of deep commercial depression and distress so unfavorable for the commencement and growth of enterprise, requiring outlay and expenditure, I can only say, that since we have been deprived of our home organ in medicine, so long and ably conducted, we have better appreciated its value and influence, and most deeply felt its loss, and though we cannot commence where it closed, in position and influence, or expect to fully compensate for its loss or removal, yet we hope to furnish an acceptable medium for the medical public, and to be taken only for "about what we are worth." While we do not deny that we are to look to the larger cities for the materials and facilities for the preparation and circulation of the more elaborate and voluminous journals, yet we claim that there are many reasons why they cannot and do not answer all the purposes and accomplish all the objects to be derived from periodical literature. That a medical journal, if it commend itself, representing fairly and fully the present advanced and growing state of medical literature, will promote and become subservient to the great mental activity, which at the present time more than ever, characterises the medical profession, will be useful in the collection and diffusion of facts, the interchange of views and opinions, not only in this section of country, but also at points remotely distant. In this enterprise we act upon the belief that the physicians whose interest, and assistance will be greatly depended upon are eminently, able and willing to contribute their proportion of effort, for the promotion and advancement of our common cause.

Our pages will be open for the expression of opinions and free discussion upon all subjects involved in the interest or progress of medicine and surgery, and an earnest and cordial invitation is hereby extended to the Profession to contribute to our pages. We shall be desirous of appropriating as much space as our limits will allow to original articles. Important cases interesting from their rarity, novelty, or the results of treatment, which often serve to develop or confirm very important truths, are constantly being treated by observing practical men. It is desirable to collect a great number of thoughts and ideas which originate in the minds of observing practitioners, placing them in the great store-house of medicine literature for analysis and comparison, to be estimated and produce results according to their intrinsic value. For the purpose of supplying condensed intelligence of discoveries, improvements and new views in medicine, surgery and the collateral sciences, we shall carefully select from both foreign and domestic journals, using their contents as opportunity or interest may indicate, not with any view to supercede the importance or necessity of resorting to them more directly, or with any idea that the contents of these journals are not very early made familiar to many of our readers, but rather to extend an appreciation of their value, and promote their more general circulation.

The editor desires to say that he is looking to the profession for the means of success in this undertaking, and will not allow himself alone to be held responsible for its success or failure, since he is depending not so much upon his own exertions as the assistance he expects to receive from others. He enters upon the duty stimulated by the hope and expectation of so complete success as soon to justify the enlargement of the Journal, rendering it more acceptable and useful than it can possibly become in its present restricted limits.

